# Pure-quartic solitons

**DOI:** 10.1038/ncomms10427

**Published:** 2016-01-29

**Authors:** Andrea Blanco-Redondo, de Sterke C. Martijn, J.E. Sipe, Thomas F. Krauss, Benjamin J. Eggleton, Chad Husko

**Affiliations:** 1 Centre for Ultrahigh bandwidth Devices for Optical Systems (CUDOS), Institute of Photonics and Optical Science (IPOS), School of Physics, The University of Sydney, Sydney, New South Wales 2006, Australia; 2 Department of Physics, University of Toronto, 60 Street George Street, Toronto, Ontario, Canada M5S 1A7; 3 Department of Physics, University of York, York, YO10 5DD, UK

## Abstract

Temporal optical solitons have been the subject of intense research due to their intriguing physics and applications in ultrafast optics and supercontinuum generation. Conventional bright optical solitons result from the interaction of anomalous group-velocity dispersion and self-phase modulation. Here we experimentally demonstrate a class of bright soliton arising purely from the interaction of negative fourth-order dispersion and self-phase modulation, which can occur even for normal group-velocity dispersion. We provide experimental and numerical evidence of shape-preserving propagation and flat temporal phase for the fundamental pure-quartic soliton and periodically modulated propagation for the higher-order pure-quartic solitons. We derive the approximate shape of the fundamental pure-quartic soliton and discover that is surprisingly Gaussian, exhibiting excellent agreement with our experimental observations. Our discovery, enabled by precise dispersion engineering, could find applications in communications, frequency combs and ultrafast lasers.

## Abstract (web summary)

Optical solitons are pulses that propagate undistorted. Here, the authors demonstrate a class of soliton arising from the interaction of self-phase modulation with quartic dispersion, rather than with quadratic dispersion as occurs in conventional solitons.

## Graphical abstract

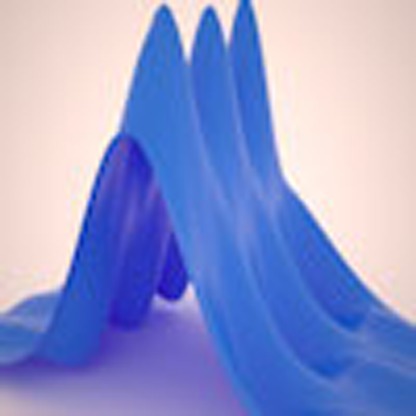

The fascinating phenomenon of optical solitons, solitary optical waves that propagate in a particle-like fashion over long distances^1^, has been the subject of intense research during the last decades due to its major role in breakthrough applications such as mode locking^2^, frequency combs^3^,^4^ and supercontinuum generation^5^,^6^ among others^7^,^8^,^9^. Temporal solitons in optical media^10^,^11^, as studied to date, arise from the balance of the phase shift due to anomalous quadratic group-velocity dispersion (GVD), that is, negative GVD parameter *β*_2_=(*∂*^2^*k*/*∂ω*^2^)<0, and the self-phase modulation (SPM) due to the nonlinear Kerr effect.

In practice, higher-order nonlinear and dispersive effects often perturb this behaviour. In silicon (semiconductor) waveguides the most significant higher-order nonlinearities are associated with free carriers (FCs) generated by two-photon absorption (TPA)^12^,^13^, which have hampered the observation of soliton-based effects in this material. Recently, some of us achieved higher-order soliton compression of picosecond pulses in silicon^14^ by using a dispersion engineered photonic crystal waveguide (PhC-wg). Turning to higher-order dispersive effects, the presence of third order dispersion (TOD; *β*_3_=*∂*^3^*k*/*∂ω*^*3*^) leads to soliton instability^15^, whereas positive fourth-order dispersion (FOD; *β*_4_=(∂^4^*k*/*∂ω*^4^)>0), can give rise to radiation at specific frequencies^16^. In the presence of negative FOD (*β*_4_<0), as was shown by a series of theoretical works in optical fibres^17^,^18^,^19^,^20^,^21^, solitons can be stable. These studies^17^,^18^,^19^,^20^,^21^ led to the concept of quartic solitons^22^, solitary pulses resulting from the interaction of anomalous GVD and SPM but modified by the presence of FOD.

In the following we report the experimental discovery and physical description of an entirely new class of solitons originating purely from the interaction of negative FOD and SPM, as conceptually depicted in Fig. 1a, which can occur even when the GVD vanishes or is normal. Since they arise just from quartic dispersion and SPM, and to distinguish them from the solitary waves studied earlier^17^,^18^,^19^,^20^,^21^,^22^, we propose the name of pure-quartic soliton for this new class of solitary wave. This experimental discovery is enabled by the unique dispersion properties of PhC-wgs, which allow us to combine very large negative *β*_4_ with small positive *β*_2_ and negligible *β*_3_ for the wavelength under study. Though our work directly pertains to pulse propagation in guided wave structures, the same ideas apply to spatial solitons, particularly to subdiffractive matter-wave solitons in regimes where the fourth-order diffraction is the dominant diffractive effect^23^,^24^. Furthermore, it was shown that in Ti:sapphire lasers FOD ultimately limits the minimum pulse duration in cavities with near-zero GVD and TOD^25^,^26^,^27^, hinting that the pulse shaping behaviour in the laser cavity for ultrashort pulses (below 10 fs) arises from the balance of SPM and FOD^28^.

## Results

### Experimental signatures of pure-quartic solitons

**Figure 1:**
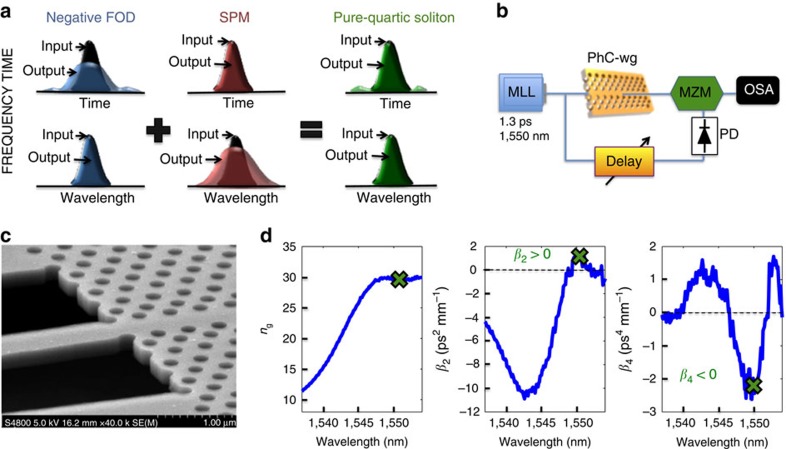
Concept of pure-quartic solitons and their experimental demonstration. (**a**) Schematics of pure-quartic solitons: (Left) Fourth-order dispersion (FOD) gives rise to temporal pulse broadening (blue output pulse versus black input pulse in time) without affecting the spectrum; (Centre) self-phase modulation (SPM) generates spectral broadening (red output pulse versus black input pulse in frequency) without affecting the temporal pulse shape; (Right) the interplay of FOD and SPM can give rise to pure-quartic solitons which remain nearly unperturbed (green output pulses versus black input pulses in both frequency and time); (**b**) Frequency-resolved electrical gating set-up: mode locked laser (MLL), photonic crystal waveguide (PhC-wg), tunable delay, ultrafast photodiode (PD), Mach–Zehnder modulator (MZM), and optical spectrum analyser (OSA); (**c**) Scanning electron microscope image of the sample; (**d**) Measured dispersion of the silicon photonic crystal waveguide used in our experiments: group index (*n*_g_), second-order dispersion parameter (*β*_2_) and fourth-order dispersion parameter (*β*_4_).

In our experiments we performed time- and phase-resolved propagation measurements on the sample using a frequency-resolved electrical gating (FREG) apparatus, depicted in Fig. 1b, which can be modelled using a generalized nonlinear Schrodinger equation (GNLSE). We show shape-preserving propagation and flat temporal phase for fundamental pure-quartic solitons and temporal compression and convex nonlinear phase for higher-order pure-quartic solitons. In spite of maintaining these well-known signatures of soliton-like behaviour, we show that pure-quartic solitons present remarkably different properties than solitons studied to date^10^,^11^,^17^,^18^,^19^,^20^,^21^. Importantly, the energy scaling of pure-quartic solitons suggests much higher energies for ultrashort pulses, which may inspire a new wave of soliton laser developments. Finally, we derive the approximate shape of fundamental pure-quartic solitons and find that it is close to a Gaussian, which is remarkable given that the solitary wave solutions found to date are always a function of hyperbolic secant^10^,^18^,^20^.

For demonstrating the existence of pure-quartic solitons we used the 396-μm-long dispersion engineered slow-light silicon PhC-wg^29^ shown in Fig. 1c (see the Methods section). Figure 1d shows the waveguide dispersion measured using an interferometric technique^30^. At the pulse central wavelength, 1,550 nm, the PhC-wg has a measured group index of *n*_g_=30, a GVD of *β*_2_=+1 ps^2^ mm^−1^, corresponding to normal dispersion, a TOD of *β*_3_=+0.02 ps^3^ mm^−1^, and a FOD of *β*_4_=−2.2 ps^4^ mm^−1^. Note that *β*_4_ is negative in a 6-nm wavelength range.

**Figure 2:**
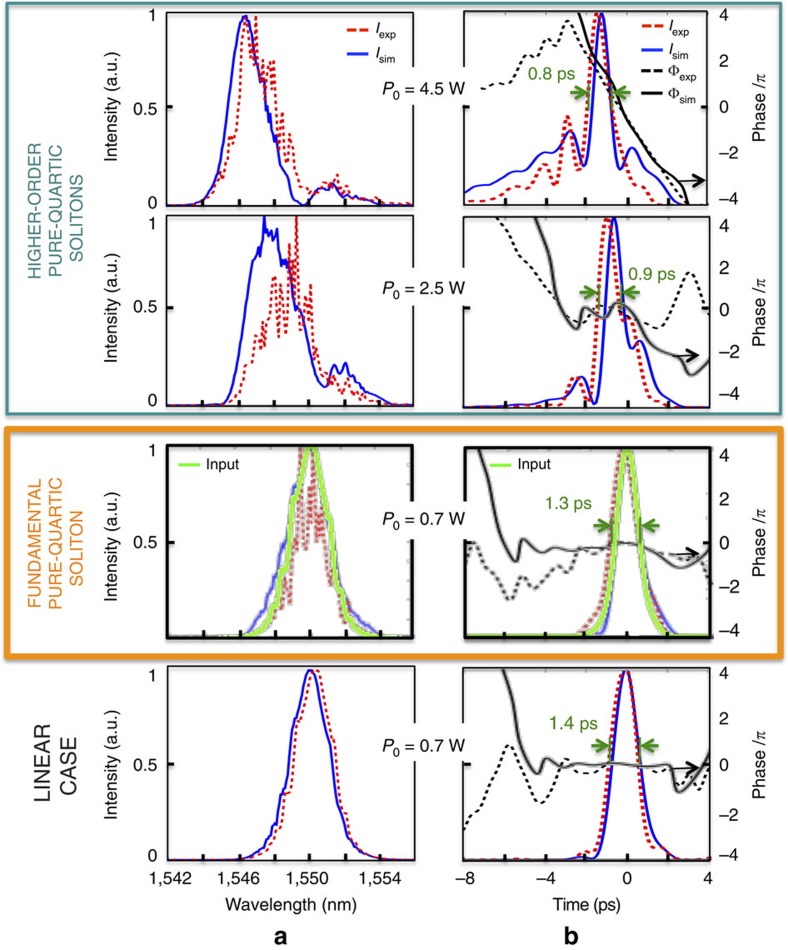
Experimental and modelling results. (**a**) Frequency and (**b**) time domain results for different input powers. The dashed red lines represent the intensity measurements, the blue solid lines represent the intensity simulations, the black dashed line represents the measured phase, and the solid black line represents the simulated phase. The green solid line at 0.7 W represents the normalized input intensity. The yellow box encompasses the fundamental pure-quartic soliton, showing nearly unperturbed propagation and flat temporal phase. The turquoise box includes two cases of higher-order pure-quartic solitons, showing temporal compression and nonlinear spectral broadening. The higher-order pure-quartic solitons observed here are greatly perturbed by the presence of free carriers.

To perform a complete temporal and spectral characterization of the sub-picojoule ultrafast nonlinear dynamics in the waveguide we used a FREG apparatus^31^ in a cross-correlation configuration, as schematically depicted in Fig. 1b (see the Methods section). This set-up provides a series of spectrograms, that is, the gated optical power versus delay, for varying input powers. From these spectrograms we then extract the optical pulses' electric field envelope and phase using a numerical algorithm^32^. Figure 2 shows the measured intensity (red dashed lines) and phase (black dashed) at the output of the PhC-wg, when injecting 1.3 ps Gaussian pulses (full-width at half-maximum, FWHM) at 1,550 nm with different input peak powers, *P*_0_. Figure 2a shows the frequency domain and Fig. 2b shows the temporal domain. The physical length scales of the dispersion orders for this pulse duration are: *L*_GVD_=*T*_0_^2^/|*β*_2_|=0.615 mm, *L*_TOD_=*T*_0_^3^/|*β*_3_|=22.6 mm, *L*_FOD_=*T*_0_^4^/|*β*_4_|=0.168 mm, with *T*_0_=FWHM/1.665 for Gaussian pulses. These length scales indicate that FOD is dominant, with the total length of the sample being *L*=2.4.*L*_FOD_ and the GVD length being *L*_GVD_=3.66.*L*_FOD_. TOD is negligible in this sample for our pulses.

To understand the origin of the experimental observations in Fig. 2 we employ a GNLSE model to describe the propagation in the silicon PhC-wg:


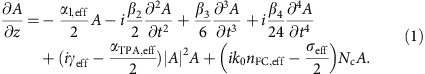


Here *A*(*z*,*t*) is the slowly varying amplitude of the pulse, *α*_l,eff_ denotes the linear loss, *γ*_eff_ and *α*_TPA,eff_ are the effective nonlinear Kerr and TPA parameters, respectively; *n*_FC,eff_ and *σ*_eff_ represent the free-carrier dispersion (FCD) and the free-carrier absorption effective parameters for a free-carrier concentration of *N*_c_. Since we use a slow-light PhC-wg, the effective coefficients vary with the slow-down factor *S*=*n*_g_/*n*_0_ (ref. 13). We use the measured envelope amplitude of the input pulse as the input to our GNLSE model with the sample parameters detailed in the Methods section. As shown in Fig. 2, the model (solid lines) agrees well with the experimental data (dashed lines) in both frequency and time.

We first focus on the time domain results of Fig. 2b. At the low coupled power of 0.07 W, nonlinear effects can be neglected and we simply observe small temporal broadening, mainly due to quartic dispersion. The different signs of *β*_2_ and *β*_4_ counteract each other to some degree, leading to a modest temporal broadening at the output of this short PhC-wg (from 1.3 to 1.4 ps). We have verified, by running the GNLSE for longer lengths that the pulse width keeps increasing with the propagation distance in the linear case. Increasing the input power up to 0.7 W, where the nonlinear length *L*_NL_=1/(*γ*_eff_*P*_0_) becomes comparable to *L*_FOD_, the pulse preserves its initial shape and duration, as illustrated by the good matching between the measured output intensity (dashed red line) and the normalized input intensity (green solid line). Furthermore, the temporal phase across the pulse duration is nearly flat. These are two signatures of fundamental soliton behaviour^10^. Simple estimates, confirmed with GNLSE simulations, show that the loss due to TPA at this power level is quite small and that free carriers do not yet play a role. At 2.5 W, where *L*_NL_<<*L*_FOD,_ the phase becomes convex due to the stronger nonlinear Kerr effect and the main peak of the pulse narrows, temporal signatures of a higher-order soliton. At even higher powers, 4.5 W, the main peak of the pulse narrows even more, corresponding to a higher-order soliton with a higher-soliton number^11^. In addition, a long tail develops towards the leading edge of the pulse. We provide an explanation for this effect below.

Next we examine the frequency domain in Fig. 2a. At 0.07 W, since the nonlinearities are negligible and the pulse spectrum is not affected by the dispersion, the pulse spectral shape is maintained. At 0.7 W the pulse preserves its initial spectral shape, again consistent with fundamental soliton behaviour in the spectral domain. At higher powers (2.5 and 4.5 W) the pulse experiences spectral broadening and splits into two peaks, spectral signatures of higher-order solitons. The observed blue shift and asymmetry are associated mainly with FCD. Note that the oscillations in the measured spectra simply correspond to Fabry–Perot reflections at the input and output facets of the PhC-wg and disorder in the periodic media^33^.

Whereas we previously reported shape-preserving fundamental solitons and higher-order soliton compression in silicon^14^, such behaviour was unforeseen for the normal GVD here. By setting *β*_2_=0 in our numerical model we find that the signatures of soliton behaviour are maintained: the shape is preserved and the phase is flat for the fundamental soliton at 0.7 W, and at 2.5 and 4.5 W, the higher-order solitons undergo nonlinear temporal narrowing. This demonstrates that GVD is not important in this system and, since we established that TOD is also negligible, that the soliton behaviour stems purely from the interaction of FOD and SPM. Furthermore, we have verified that the long tail at the leading edge observed at high powers (Fig. 2b), as well as the self-acceleration of the pulse, originate from the interaction of negative FOD and FCD. The FCD generates additional blue components (Fig. 2a) and the negative FOD makes them travel faster than the red components of the pulse, analogous to our earlier results with negative GVD^13^,^14^,^34^.

These observations at the output of the silicon PhC-wg suggest the existence of a new type of soliton: pure-quartic solitons. We use the term soliton here to refer to solitary optical waves that propagate essentially unperturbed over long distances, not to exact localized solutions of integrable nonlinear differential equations^35^.

As expected in a silicon system at 1,550 nm, pure-quartic solitons are strongly perturbed by TPA and FC as we just described, and thus the measured behaviour differs from the simple case with just SPM and FOD. Therefore, to elucidate the dynamics of pure-quartic solitons in the absence of higher-order nonlinearities we next numerically study the propagation of picosecond pulses along the PhC-wg neglecting all effects but SPM and FOD.

### Propagation behaviour of pure-quartic solitons

**Figure 3:**
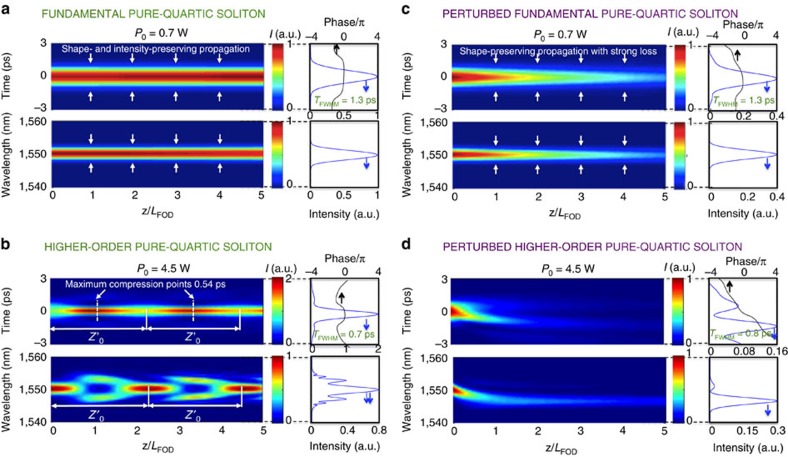
Simulations of the propagation of a fundamental and a higher-order pure-quartic soliton along five quartic dispersion lengths, *L*_FOD_. (**a**) Fundamental (*P*_0_*=*0.7 W) pure-quartic soliton with only self-phase modulation and quartic dispersion present, and (**c**) in the more realistic scenario for our silicon waveguide with two-photon absorption and free carriers; (**b**) and (**d**) are similar but for a higher power level (*P*_0_*=*4.5 W) where a higher-order pure-quartic soliton results.

Figure 3a,b depict the propagation dynamics of undistorted pure-quartic solitons, that is, in the presence of SPM and FOD only. Such a system is governed by the biharmonic nonlinear Schrodinger equation


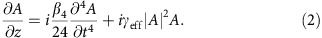


In our simulations we consider two different power levels: fundamental pure-quartic solitons occur at moderate powers (Fig. 3a), whereas at high powers higher-order pure-quartic solitons result (Fig. 3b).

The simulations in Fig. 3a show shape-preserving pulse propagation in time and frequency over five quartic dispersion lengths *L*_FOD_ for a fundamental pure-quartic soliton. The very slight increase in the maximum intensity at *t*=0 corresponds to the pulse adapting itself from the standard Gaussian pulse used as an input to the model, to the soliton form whose approximate shape we provide in the next section. The output, represented by the blue curve to the right of the propagation plot, shows that the pulse maintains essentially the same amplitude, shape (Gaussian), and duration (1.3 ps) as the input pulse. Importantly, the temporal phase at the output, represented by the black solid line, is flat across the duration of the pulse.

Figure 3b reveals a higher-order pure-quartic soliton, with the pulse experiencing periodic recurrent propagation. In time the pulse undergoes compression and then periodically returns to its initial shape. In frequency the pulse splits into two and then recombines to recover its initial spectral shape after the same period. At the maximum compression point, the pulse reaches the minimum duration of 0.54 ps, a compression factor of 2.4 compared with the initial pulse duration, with a peak intensity of two times that of the initial pulse. Our simulations show the compression factor of the pure-quartic soliton roughly follows the same trend as conventional solitons^11^. Specifically, larger intensities lead to higher compression factors and to compression occurring at an earlier spatial position along the waveguide. Crucially, while the trends are superficially similar to the behaviour of conventional solitons, the different scaling of SPM and FOD with pulse length suggests that the well-known definitions of soliton number and soliton period will not be appropriate for pure-quartic solitons; further studies are underway to derive the appropriate parameters and physical scaling laws for these field structures.

To understand why the experimental observations of pure-quartic solitons in Fig. 2 differ from the numerical results of the undistorted system in Fig. 3a,b, we simulate the propagation along the PhC-wg including all the effects in the real system indicated in equation 1. The outputs shown in Fig. 3c,d match our experimental measurements at *P*_0_=0.7 W and *P*_0_=4.5 W. Figure 3c shows that the signatures of the fundamental pure-quartic soliton remain in realistic simulations: the pulse maintains its shape and width, and the phase at the output remains almost flat. However, the intensity of the pulse decreases due, predominantly, to the linear loss in the slow-light waveguide ∼70 dB cm^−1^. Since the intensity decreases as the pulse propagates, the linear FOD will eventually dominate over the SPM for longer distances, leading to temporal broadening of the fundamental pure-quartic soliton. The higher-order pure-quartic soliton in the realistic scenario of Fig. 3d differs considerably from Fig. 3b. The TPA clamps the intensity in the waveguide from the early stages of propagation. The FCD introduces blue components that lead to the self-acceleration of the pulse and an asymmetry, and the free-carrier absorption induces absorption on the trailing edge. These effects of TPA and FCs on the propagation of higher-order pure-quartic solitons in silicon are analogous to the effects of FCs on conventional solitons^14^.

### Approximate solution for the fundamental pure-quartic soliton

We now derive an analytic expression for the fundamental pure-quartic soliton. The experimental observations and numerical simulations indicate that the central part of fundamental pure-quartic solitons appears to be Gaussian. Assuming this shape, we look for an approximate solution to equation (2) in two separate ways for verification purposes: using the variational principle and looking for a local approximation near the centre. The complete derivations for the variational and local approximate solutions are described in Supplementary Note 1 and Supplementary Note 2, respectively.

In both cases, after a simple dimensional analysis, we take the central part of the pure-quartic soliton to be of the form


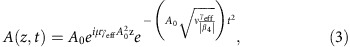


where, since *β*_4_<0, we have written 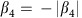
for convenience, and *μ* and *ν* are free parameters. The variational approach gives 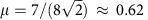, 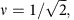
whereas the local approximation gives 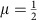, *v*=1. Thus, these approaches predict pulse widths which differ only by a factor 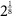 or by <10%. The fact that these different approximations give very similar results reinforces our confidence in them. Based on these results, the argument of Akhmediev and Karlsson^36^ suggests that pure-quartic solitons do not lose energy due to linear radiation. []ally, taking the Fourier transform of the right-hand side equation (3) in time and position leads to a straight line in an *ω-k* diagram. Since this straight line does not intersect the linear dispersion relation of the medium, the soliton cannot lose energy to dispersive waves.

**Figure 4:**
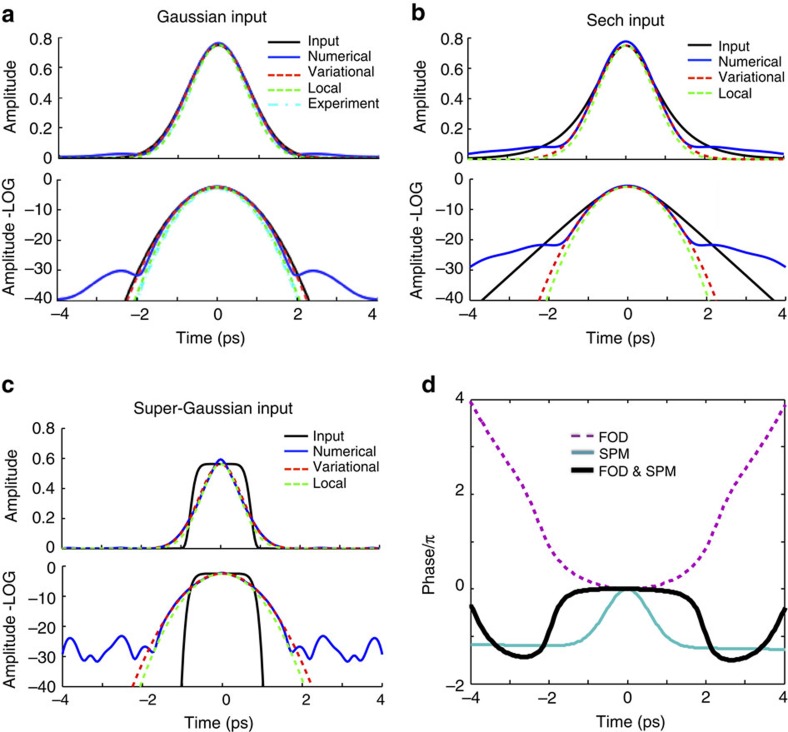
Approximate solutions to a fundamental pure-quartic soliton and phase diagram. Comparison between the variational and local approximate solutions for the fundamental pure-quartic soliton and the numerical output after propagating over thirty quartic dispersion lengths for (**a**) a Gaussian input, (**b**) a hyperbolic secant input, and (**c**) a super Gaussian input of order 4. In the Gaussian case the measured output pulse at 0.7 W is shown in the background (dot-dash cyan curve). (**d**) Phase shift induced by the fourth-order dispersion (dashed purple) and the self-phase modulation (solid turquoise) independently and its combined phase shift (black).

To test the validity of this analytic approximate solution we numerically solve equation (2) with the *β*_4_ and *γ*_eff_ of our sample (see Methods section) and obtain the output of the system at the power level corresponding to a fundamental pure-quartic soliton for a propagation length *L*=30·*L*_FOD_. This long propagation distance ensures convergence of the pulse evolution. The results of this numerical experiment for three different pulse shapes: a Gaussian, a hyperbolic secant (sech), and a super Gaussian of order four, are depicted by a solid blue curve in Fig. 4a–c, respectively. Importantly, the hyperbolic secant and super Gaussian inputs (black solid curve in Fig. 4b,c, respectively) evolve into the solitary wave Gaussian shape, constituting an additional signature of soliton-like behaviour and proving that this type of soliton acts as an attractor. These results are overlapped with the variational approximation (dashed red curve) and the local approximation (green dashed curve), using the same parameters. The agreement between the numerical and the variational solution in the central part of the pulse is remarkable. The local approximation deviates only slightly. In addition we numerically find that *μ*≈0.63, again very close to the variational result. In the background of Fig. 4a, we show the measured pulse at the output of the chip at *P*_0_=0.7 W (cyan dot-dashed curve), which matches variational solution perfectly. The wings observed in the numerical solution relate to the fact that the phase shift profile of the FOD has a quartic dependence with time, whereas the SPM varies quadratically, as illustrated in Fig. 4d. This allows both phase shift profiles to perfectly counterbalance each other close to the centre of the pulse, but deviate from each other at the edges. This fact is not captured in the approximate analytic solution since a perfect balance between FOD and SPM is assumed.

**Figure 5:**
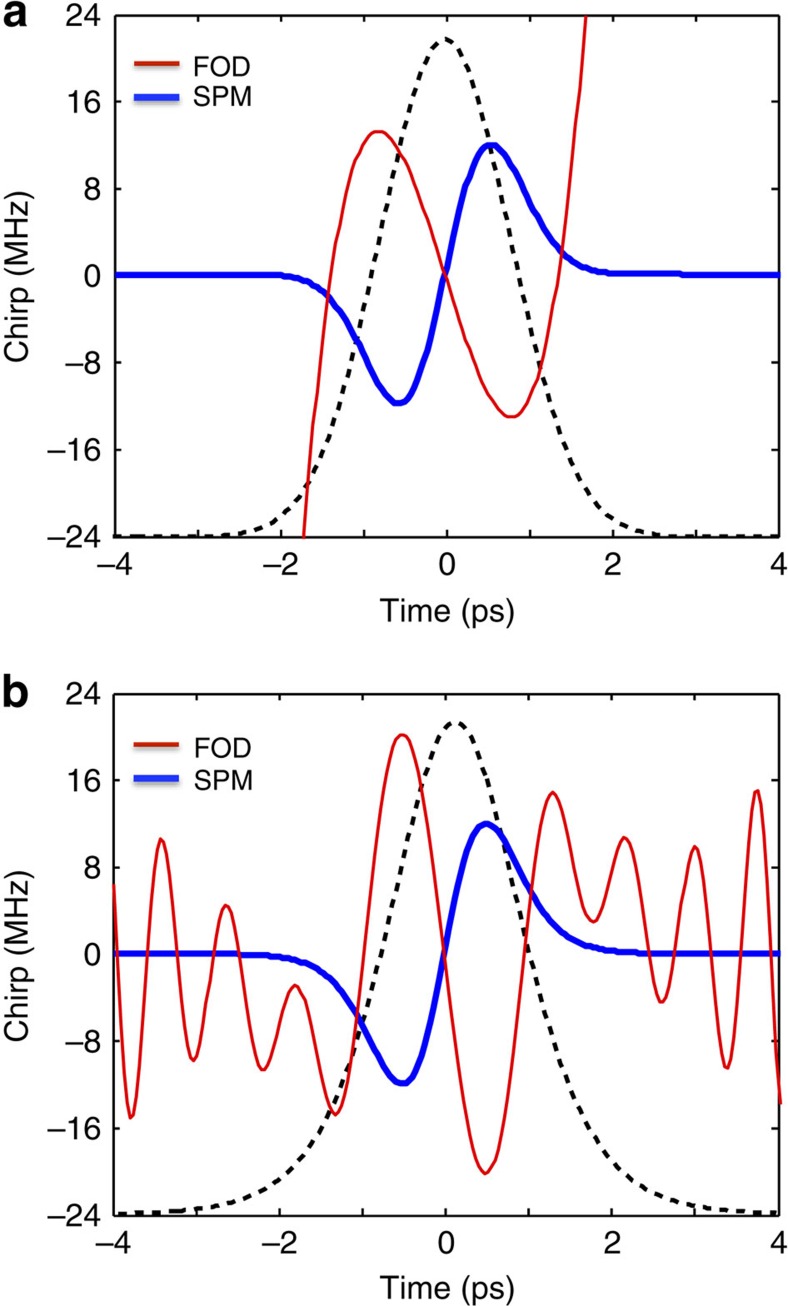
The cancellation of nonlinear and dispersive phase components on the fundamental pure-quartic soliton. (**a**) FOD-induced (red) and SPM-induced (blue) frequency chirps after a propagation of *L*_FOD_/10 for the Gaussian pure-quartic soliton of equation (3); (**b**) similar, but for the sech^2^ type solutions obtained taking ref. 18 with *β*_2_=0. The dashed black curves in the background of (**a**) and (**b**) represent the input pulse intensity: the Gaussian solution of equation 3 and the sech^2^ solution in ref. 18 respectively.

Since temporal optical solitons studied to date have some kind of hyperbolic secant shape^10^,^18^,^20^, it is surprising that pure-quartic solitons are approximately Gaussian. To understand this better, we apply the argument of Dudley *et al.*^37^ to our system, according to which the dispersive and nonlinear phase components developed during short propagation distances must cancel each other across the pulse duration to lead to the formation of a soliton. Figure 5a shows the FOD- (red) and the SPM-induced chirp (blue) after propagating the Gaussian variational solution in equation 3 (dashed black curve) for a distance *L*_FOD_/10 and demonstrates how this solution leads to the mentioned cancellation across the central part of the pulse. To highlight the different nature of the solutions found here with respect to the previously studied NLS solitons with FOD, we apply the same verification to the solution found in ref. 18, in the limit *β*_2_=0. Figure 5b shows how the sech^2^ solution obtained from ref. 18 (dashed black curve) does not provide the necessary cancellation of the SPM- and FOD-induced chirp required for the formation of a stable solitary wave in the presence of just SPM and FOD.

## Discussion

The experimental results and the numerical simulations presented here have established the existence of a new class of solitons: pure-quartic solitons, arising from the interaction of SPM and FOD only. In particular, we experimentally demonstrated shape-preservation and flat-phase behaviour for the fundamental pure-quartic soliton, and temporal compression for the higher-order pure-quartic solitons. We numerically demonstrated that the higher-order pure-quartic soliton would undergo recurrent periodic propagation in the absence of loss and higher-order nonlinearities. Although we have verified that these signatures of soliton propagation are preserved for long propagation distances in the presence of just FOD and SPM, the disparity between the quartic profile of the FOD-induced phase shift and the quadratic profile of the SPM-induced phase shift affecting the edges of the pulse could lead to stability issues that should be studied. Establishing appropriate definitions of concepts such as soliton number and soliton period for pure-quartic solitons is an open theoretical challenge.

Our discovery was facilitated by the unique dispersion properties of PhC-wgs that provide the design freedom to achieve a wide variety of dispersion profiles. However, other guided wave systems such as highly nonlinear fibres^38^, photonic crystal fibres^8^ or specially designed silicon waveguides^22^,^39^, could also be engineered to observe pure-quartic solitons. The main condition to fulfil is *L*_FOD_<<*L*_GVD_, *L*_TOD_ (with *β*_4_<0), and, in practice, we have verified that *L*_FOD_<<*L*_GVD_/3 across most of the pulse bandwidth is enough for a robust observation. The pure-quartic soliton behaviour starts to become observable when *L*_FOD_ becomes comparable to the sample length. However, our initial simulations show that the pure-quartic soliton does not reach a steady state until it propagates for several quartic dispersion lengths, similar to conventional solitons^40^. Analogous regimes of evolution have been demonstrated in Ti:sapphire laser cavities^25^,^26^,^27^,^28^, taking advantage of the rich variety of physical regimes offered by the discrete structure of the laser cavity. For example, Zhou *et al.* demonstrated in ref. 26 8.5 fs pulses from a Ti:sapphire laser operating near zero GVD with the minimum pulse duration limited by FOD, and later Christov *et al.*^28^ hinted that the ‘soliton-like pulse' inside such a laser was ‘fourth-order dispersion limited'. Here we experimentally demonstrate that the balance between SPM and FOD gives rise to robust soliton-like behaviour. Hence, the scope of our findings is not just limited to nonlinear guided wave optics, but may provide novel insights into extreme regimes of ultrafast lasers operation.

The Gaussian variational solution provided here constitutes a good approximation to the central form of the fundamental pure-quartic soliton. The results of our study on the cancellation of the nonlinear and quartic dispersion phase components in short propagation distances proved that no previously found solitary wave solution^10^,^18^,^20^ can describe the behaviour of pure-quartic solitons. This approximate solution, valid only for *β*_4_<0, could stimulate new efforts in finding solutions to the biharmonic nonlinear Schrodinger equation^41^,^42^ also of interest in the field of spatial solitons^23^,^24^,^43^,^44^. Recent interest in temporal cavity solitons in both microresonators^3^ and optical fibres^7^ with applications in Kerr frequency combs^45^ and low-noise microwave generation^46^ could also benefit from exploring pure-quartic solitons in their systems. Furthermore, it would be interesting to investigate analytic solutions supported by the pure-quartic soliton system including the effects of linear loss, TPA and FCs.

Aside from their different physical origin, pure-quartic solitons present significant potential advantages with respect to conventional solitons. As mentioned, pure-quartic solitons open the door to soliton functionality in the normal GVD regime of optical media. More importantly, perhaps, the energy of conventional solitons scales like (*T*_0_)^−1^, whereas the energy of pure-quartic solitons scales like (*T*_0_)^−3^, which suggests that they are more energetic for ultrashort pulses. We expect that the understanding of pure-quartic solitons provided in this paper, combined with the previous advances in the laser literature^25^,^26^,^27^,^28^, will inspire a new wave of ultrafast laser development.

## Methods

### Device and linear characterization

The present experiment was performed using a silicon photonic crystal air-suspended structure with a hexagonal lattice (p6m symmetry group) constant *a=*404 nm, a hole radius *r=*116 nm, and a thickness *t=*220 nm. A 396-μm-long dispersion engineered PhC-wg was created by removing a row of holes and shifting the two innermost adjacent rows 50 nm away from the line defect. The air-clad devices were fabricated with a combination of electron beam lithography, reactive ion and chemical wet etching. The measured linear propagation loss in this slow-light region is ∼70 dB cm^−1^, with a total linear insertion loss of ∼13 dB (5 dB per facet). Light was coupled in with tapered lensed fibres to SU8 polymer waveguides with inverse tapers.

### Phase-resolved characterization method

For the nonlinear experiments, we used a mode locked laser (Alnair) fed into a pulse shaper (Finisar) generating near transform-limited 1.3 ps pulses at 1,550 nm at a 30 MHz repetition rate. These pulses were then input into the FREG apparatus. The pulses were split into two branches by a fiber-coupler, with the majority of the energy coupled into the PhC-wg. The remaining fraction was sent to a reference branch with a variable delay, before being detected by a fast photodiode and transferred to the electronic domain. This electronic signal drove a Mach–Zender modulator that gated the optical pulse output from the PhC-wg. Using an optical spectrum analyser, we measured the spectra as a function of delay to generate a series of optical spectrograms. We de-convolved the spectrograms with a numerical algorithm (256 × 256 grid-retrieval errors *G*<0.005), to retrieve the pulse intensity and the phase in both the temporal and spectral domain^32^.

### Generalized nonlinear Schodinger equation model

The parameters used in our GNLSE model for the slow-light dispersion engineered PhC-wg are: slow-down factor *S*=*n*_g_/*n*_0_=8.64, effective linear absorption *α*_l,eff_*=*13.9 cm^−1^ ; *β*_2_=+1 ps^2^  mm^−1^, a TOD parameter of *β*_3_=0.02 ps^3^ mm^−1^, and a FOD parameter of *β*_4_=−2.2 ps^4^ mm^−1^; effective nonlinear parameter 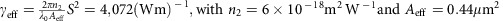; effective TPA parameter 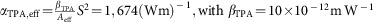; effective free-carrier dispersion parameter *n*_FC,eff_=−6 × 10^−27^ S m^3^; effective free-carrier absorption parameter *σ*_eff_=1.45 × 10^−21^ S m^2^. The simulation results in Fig. 2 were obtained by using the measured input pulse as the input to the model. The simulation results in Fig. 4 were obtained using a perfect Gaussian, hyperbolic secant, and a super Gaussian (order 4) pulse of the same width as the experimental pulse, 1.3 ps. The linear loss in the nanowires that couple light into and out of the PhC-wg was negligible. Nonlinear absorption in the coupling nanowire (effective area, ∼0.2 μm^2^) was taken into account in the NLSE model.

## Additional information

**How to cite this article:** Blanco-Redondo, A. *et al.* Pure-quartic solitons. *Nat. Commun.* 7:10427 doi: 10.1038/ncomms10427 (2016).

## Supplementary Material

Supplementary Information Supplementary Notes 1-2.
