# Erratum: Pure-quartic solitons

**DOI:** 10.1038/ncomms11048

**Published:** 2016-03-09

**Authors:** Andrea Blanco-Redondo, C. Martijn de Sterke, J. E. Sipe, Thomas F. Krauss, Benjamin J. Eggleton, Chad Husko

Nature Communications 7: Article number: 10427 ; DOI: 10.1038/ncomms10427 (2016); Published: 01 29 2016; Updated: 03 09 2016

The original version of this article contained an error in the spelling of the author C. Martijn de Sterke, which was incorrectly given as de Sterke C. Martijn. This has now been corrected in both the PDF and HTML versions of the article.

