# Supplementary material for: Pure-quartic solitons
Source: Nat Commun. 2016 Jan 29;7:10427. doi: 10.1038/ncomms10427 (PMC4740180; doi:10.1038/ncomms10427)
Supplement: Supplementary Information — Supplementary Notes 1-2. [file ncomms10427-s1.pdf]

Supplementary Notes

In the following notes we derive an analytic expression approximately describing the canonical form of a *fundamental pure-quartic soliton*. The canonical system, in the presence of just negative fourth-order dispersion (FOD), i.e. β_4<0, and self-phase-modulation (SPM) is described by the equation

$$i\frac{\partial A}{\partial z} = \frac{|\beta_4|}{24}\frac{\partial^4 A}{\partial t^4} - \gamma_{eff}|A|^2 A. \qquad (1)$$

The experimental observations and numerical simulations previously presented in this paper indicate that a Gaussian is a good approximation to the form of the *fundamental pure-quartic soliton*. Next, we look for an approximate solution to Eq. (1) in two separate ways: using the variational principle and looking for a local approximation.

Supplementary Note 1

Here we derive the variational approximation to fundamental pure-quartic-solitons. Assuming that a Gaussian is a good approximation, we can consider a related problem defined by the eigenvalue equation:

$$\mathcal{H}(t)\psi(t) = E\psi(t), \qquad (2)$$

where the Hamiltonian operator $\mathcal{H}(t)$ is given by

$$\mathcal{H}(t) = \frac{|\beta_4|}{24}\frac{\partial^4}{\partial t^4} - \gamma_{eff}A_0^2 e^{-2\sigma t^2} \qquad , \qquad (3)$$

with A_0 and σ being real and positive numbers, E being the eigenvalue, and $\psi(t)$ a given wave function. The exponential factor with σ is the nonlinear refractive index profile of the soliton, but we do not use this information yet. Since $\mathcal{H}(t)$ is a Hermitian operator we can look for an approximate solution to Eq. (2) using the variational principle. We pick a trial wave function of the form

$$\psi(t) \propto e^{-\delta t^2}, \qquad (4)$$

where δ is the variational parameter we will adjust. The expectation value of the energy term is then

$$E(\delta) = \frac{\frac{|\beta_4|}{24}\int \psi^*(t)\frac{\partial^4}{\partial t^4}\ \psi(t)dt - \gamma_{eff}A_0^2\int e^{-2\sigma t^2}\ |\psi(t)|^2 dt}{\int |\psi(t)|^2 dt}. \qquad (5)$$

Doing two partial integrations, and taking into account that $\psi(t)$ is real, Eq. (5) can be simplified to

$$E(\delta) = \frac{\frac{|\beta_4|}{24}\int \left(\frac{\partial^2\psi(t)}{\partial t^2}\right)^2 \psi(t)dt - \gamma_{eff}A_0^2\int e^{-2(\sigma+\delta)t^2}dt}{\int e^{-2\delta t^2}dt}, \qquad (6)$$

where we have used the fact that the result is independent of the normalization of $\psi(t)$, so we can take $\psi(t) = e^{-\delta t^2}$. Substituting $\frac{\partial^2\psi(t)}{\partial t^2}$ in Eq. 6, we get

$$E(\delta) = \frac{\frac{|\beta_4|}{24}\left(4\delta^2\int e^{-2\delta t^2}dt + 16\delta^4\int t^4 e^{-2\delta t^2}dt - 16\delta^3\int t^2 e^{-2\delta t^2}dt\right) - \gamma_{eff}A_0^2\int e^{-2(\sigma+\delta)t^2}dt}{\int e^{-2\delta t^2}dt}. \qquad (7)$$

Evaluating the integrals we find

$$E(\delta) = \frac{|\beta_4|\delta^2}{8} - \gamma_{eff} A_0^2 \frac{\delta^{\frac{1}{2}}}{(\delta+\sigma)^{\frac{1}{2}}} \,. \tag{8}$$

According to the variational principle, for fixed A_0 and σ, i.e. for fixed Hamiltonian $\mathcal{H}(t)$, the best solution of the form we have adopted will be found by forcing $\frac{dE(\delta)}{d\delta} = 0$. This yields

$$\frac{dE(\delta)}{d\delta} = \frac{|\beta_4|\delta}{4} - \frac{1}{2}\gamma_{eff} A_0^2 \frac{1}{\delta^{\frac{1}{2}}(\delta+\sigma)^{\frac{1}{2}}} + \frac{1}{2}\gamma_{eff} A_0^2 \frac{\delta^{\frac{1}{2}}}{(\delta+\sigma)^{\frac{3}{2}}} = 0, \tag{9}$$

that determines the δ value that minimizes the energy of the trial wave function, i.e. the "best δ". However, we are interested in knowing, under which circumstances the "best δ" equals σ. By setting $\delta = \sigma$ in Eq. (9) we get to

$$\sigma^2 = \frac{\gamma_{eff}}{\sqrt{2}|\beta_4|} A_0^2, \tag{10}$$

which determines the conditions under which the "best δ" equals σ. In this special case, our estimate for the energy (8) is

$$E(\delta) = -\frac{7}{8}\frac{\gamma_{eff} A_0^2}{\sqrt{2}}. \tag{11}$$

Using (3), (10) and (11) we can write a special case of Eq. (2) as

$$\left(\frac{|\beta_4|}{24}\frac{\partial^4}{\partial t^4} - \gamma_{eff} A_0^2 e^{-2\left(A_0\sqrt{\frac{\gamma_{eff}}{\sqrt{2}|\beta_4|}}\right)t^2}\right)\left(A_0 e^{-\left(A_0\sqrt{\frac{\gamma_{eff}}{\sqrt{2}|\beta_4|}}\right)t^2}\right) \approx \left(-\frac{7}{8}\frac{\gamma_{eff} A_0^2}{\sqrt{2}}\right)\left(A_0 e^{-\left(A_0\sqrt{\frac{\gamma_{eff}}{\sqrt{2}|\beta_4|}}\right)t^2}\right) \tag{12}$$

Next we return to Eq. (1) and look for an approximate solution of the form:

$$A(z,t) = A_0 e^{i\Gamma z} e^{-\left(A_0\sqrt{\frac{\gamma_{eff}}{\sqrt{2}|\beta_4|}}\right)t^2}. \tag{13}$$

Substituting Eq. (13) in Eq. (1) we find

$$-\Gamma\left(A_0 e^{i\Gamma z} e^{-\left(A_0\sqrt{\frac{\gamma_{eff}}{\sqrt{2}|\beta_4|}}\right)t^2}\right) = \left(\frac{|\beta_4|}{24}\frac{\partial^4}{\partial t^4} - \gamma_{eff} A_0^2 e^{-2\left(A_0\sqrt{\frac{\gamma_{eff}}{\sqrt{2}|\beta_4|}}\right)t^2}\right)\left(A_0 e^{i\Gamma z} e^{-\left(A_0\sqrt{\frac{\gamma_{eff}}{\sqrt{2}|\beta_4|}}\right)t^2}\right). \tag{14}$$

Using Eq. (12) and Eq. (14) we find

$$-\Gamma\left(A_0 e^{i\Gamma z} e^{-\left(A_0\sqrt{\frac{\gamma_{eff}}{\sqrt{2}|\beta_4|}}\right)t^2}\right) \approx \left(-\frac{7}{8}\frac{\gamma_{eff} A_0^2}{\sqrt{2}}\right)\left(A_0 e^{i\Gamma z} e^{-\left(A_0\sqrt{\frac{\gamma_{eff}}{\sqrt{2}|\beta_4|}}\right)t^2}\right), \tag{15}$$

which yields

$$\Gamma = \frac{7}{8}\frac{\gamma_{eff} A_0^2}{\sqrt{2}}. \tag{16}$$

We finally get to a class of approximate solutions to Eq. (1), characterized by their maximum input amplitude A_0 of the form

$$A(z,t) = A_0 e^{i\left(\frac{7}{8}\frac{\gamma_{eff}A_0^2}{\sqrt{2}}\right)z} e^{-\left(A_0\sqrt{\frac{\gamma_{eff}}{\sqrt{2}|\beta_4|}}\right)t^2} \quad . \tag{17}$$

Supplementary Note 2

Here, we find an approximate solution to Eq. (1) using a local approximation. Since we know a Gaussian is a good approximation we take

$$A(z,t) = A_0 e^{i\Gamma z} e^{-\frac{t^2}{\tau^2}}, \tag{18}$$

which is equivalent to Eq. (3) in the main text. Substituting Eq. (18) in Eq. (1) and Taylor expanding $e^{-\frac{t^2}{\tau^2}}$ we get to

$$-\Gamma A_0 \left(1 - \frac{t^2}{\tau^2} + \frac{t^4}{2\tau^4}\right) = \frac{|\beta_4|}{24}\frac{4}{\tau^4}\left(3 - 12\frac{t^2}{\tau} + 4\frac{t^4}{\tau}\right)\left(1 - \frac{t^2}{\tau^2} + \frac{t^4}{2\tau^4}\right)A_0 - \gamma_{eff}\left(1 - 3\frac{t^2}{\tau^2} + \frac{9}{2}\frac{t^4}{\tau^4}\right)A_0^3. \tag{19}$$

Solving Eq. (19) to order t^0 and t^2, respectively, yields

$$\Gamma = \gamma_{eff}A_0^2 - \frac{1}{2}\frac{|\beta_4|}{\tau^4} \tag{20}$$

and

$$\Gamma = 3\gamma_{eff}A_0^2 - \frac{5}{2}\frac{|\beta_4|}{\tau^4}. \tag{21}$$

The intersection between Eq. (20) and Eq. (21) determines the parameters τ and Γ for this approximation

$$\tau = \sqrt[4]{\frac{|\beta_4|}{\gamma A_0^2}}, \tag{22}$$

$$\Gamma = 3\gamma_{eff}A_0^2 - \frac{5}{2}\frac{|\beta_4|}{\tau^4}. \tag{23}$$

Therefore, using a local method, we get to a class of approximate solutions to Eq. (1), characterized by their maximum input amplitude A_0 of the form

$$A(z,t) = A_0 e^{i\left(\frac{1}{2}\gamma_{eff}A_0^2\right)z} e^{-\left(A_0\sqrt{\frac{\gamma_{eff}}{|\beta_4|}}\right)t^2} \quad . \tag{24}$$

The fact that the variational and local approximations give very similar results, as illustrated by Eq. (17) and Eq. (24), together with the good matching they provide to the numerical solution of Eq. (1), reinforces our confidence in them.
